# Effects of Asymmetric Local Joule Heating on Silicon Nanowire-Based Devices Formed by Dielectrophoresis Alignment Across Pt Electrodes

**DOI:** 10.1186/s11671-017-2423-z

**Published:** 2018-01-16

**Authors:** Hsiang-Hsi Ho, Chun-Lung Lin, Wei-Che Tsai, Liang-Zheng Hong, Cheng-Han Lyu, Hsun-Feng Hsu

**Affiliations:** Department of Materials Science and Engineering, National Chung Hsing University, 145 Xingda Rd, Taichung, 40227 Taiwan

**Keywords:** Silicon, Nanowire, Dielectrophoresis, Joule heating, Photodetector

## Abstract

We demonstrate the fabrication and characterization of silicon nanowire-based devices in metal-nanowire-metal configuration using direct current dielectrophoresis. The current-voltage characteristics of the devices were found rectifying, and their direction of rectification could be determined by voltage sweep direction due to the asymmetric Joule heating effect that occurred in the electrical measurement process. The photosensing properties of the rectifying devices were investigated. It reveals that when the rectifying device was in reverse-biased mode, the excellent photoresponse was achieved due to the strong built-in electric field at the junction interface. It is expected that rectifying silicon nanowire-based devices through this novel and facile method can be potentially applied to other applications such as logic gates and sensors.

**Electronic supplementary material:**

The online version of this article (10.1186/s11671-017-2423-z) contains supplementary material, which is available to authorized users.

## Background

One-dimensional (1D) semiconductor nanowires (NWs) have attracted much attention due to their high surface-to-volume ratio, quantum confinement effect, and high crystal quality. With the tunable electrical and optical properties, Si NWs have been successfully incorporated in solar cells [1], light-emitting diodes [2], and photodetectors [3].

Several fabrication techniques have been reported for Si NWs, and these can be divided in two categories: bottom-up and top-down methods. In the bottom-up methods, atoms and molecules can be used as building blocks for the nanostructures utilizing vapor-liquid-solid (VLS) technique [4], molecular beam epitaxy (MBE) [5], or laser ablation [6]. The top-down methods including deep reactive-ion etching (DRIE) [7, 8] and metal-assisted chemical etching (MACE) [9, 10] have been introduced for nanostructures by downscaling bulk materials. Recently, a facile and high-throughput method for large-area Si NW arrays of the same dimensions has been proposed by combing MACE with nanosphere lithography (NSL) [11, 12].

Dielectrophoresis (DEP) is one of the commonly used methods applied to align NWs such as metal [13], metal oxides [14–19], Si [20–22], silicide [23], and III–V semiconductor [24] NWs for integrated devices, which were usually in metal-semiconductor-metal structures. In DEP process, the dielectric NWs are exerted by DEP forces through induced dipoles when the NWs are usually subjected to a nonuniform alternating current (AC) electric field, and therefore can precisely align across electrodes. The devices fabricated by DEP method have been investigated extensively for their electrical properties and used for many applications such as logic gates [21] and sensors [14, 16–19]. However, these devices with rectifying current-voltage (I-V) characteristics would be possibly formed in the DEP alignment. Harnack et al. [14] proposed that the factors for the rectifying behavior in the ZnO NW-based device can be attributed to dipole moment in ZnO nanocrystals with wurtzite structure or the different Schottky barrier heights on both ends of the aligned NW. Wang et al. [15] further identified that the origin of the rectifying behavior in this case could be the asymmetrical ZnO NW/Au contacts, which were generated with a different degree of annealing at the two sides in the DEP alignment.

In order to apply Si NWs on integrated devices, it is essential to understand the role of NW/metal contacts and its effect on electrical properties. Here, we demonstrate the fabrication of Si NW-based devices by direct current (DC) DEP and systematically investigate the contacts of homogeneous single-crystalized Si NWs with Pt electrodes. After an investigation of the electrical properties in these devices, we found that their I-V characteristics showed rectifying behavior and unique photosensing properties.

## Experimental

For the Si NWs fabrication method, MACE combined with NSL, reported elsewhere [11, 12], an n-type Si (100) with resistivity ranging from 1 to 10 Ω cm was cut into 1 × 1 cm^2^ pieces. The substrates were cleaned using the standard Radio Corporation of America (RCA) procedures and made hydrophilic after immersing in boiling Piranha solution, a mixture of H_2_O_2_ with H_2_SO_4_ in a ratio of 1:3, for 10 min. A close-packed monolayer of polystyrene (PS) spheres with an average diameter of 220 nm was formed on the substrates by a modified dip-coating method [25] and subsequently reduced sphere size by O_2_ plasma. A 20-nm-thick sputtered Ag thin film was deposited on the patterned substrates. The samples were etched by a mixture solution of HF, H_2_O_2_, and deionized water (HF = 5 M and H_2_O_2_ = 0.176 M) at 25 °C for 15 min. A large-area ordered Si NW arrays were obtained after removing the residual PS spheres and Ag thin film by tetrahydrofuran (THF) and HNO_3_ solution, respectively. The as-synthesized products were characterized by field emission scanning electron microscope (FESEM, JEOL, JSM-6700F) and high-solution transmission electron microscope (HRTEM, JEOL, JEM-2100F).

**Fig. 1:**
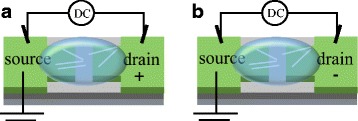
Schematics of the Si NW alignment by DC-DEP across Pt electrodes. The drain electrode was biased positively and negatively as shown in **a** and **b**, respectively. The source electrode was connected to ground level

To study the electrical transport and photosensing properties of the Si NWs, Si NW-based devices were fabricated as follows. The electrode structures were fabricated on the highly doped n-type Si (100) substrate (0.001–0.006 Ω cm) with 360-nm-thick Si oxide via traditional lithography. The electrode material was thermally evaporated Pt (40 nm)/titanium (15 nm) on top. The gap between the electrodes is about 2 μm. The as-etched Si NW arrays were removed from the substrate by 5 min of sonication and dispersed in isopropyl alcohol (IPA) solution. As shown in Fig. 1, a droplet of Si NWs suspension was dropped on top of the predefined metal electrodes applied by DC electric field. In the aligning process, the source electrode was connected to ground level while the drain one was biased positively or negatively as shown in Fig. 1.

The electrical transport properties of Si NW-based devices were conducted by the probe station using system source meter (Keithley 2612A). A broadband white light with the intensity of 825 mW/cm^2^ from an arc Hg-Xe lamp was vertically shown on the devices, and the corresponding photoresponse characteristics were recorded.

## Results and Discussion

**Fig. 2:**
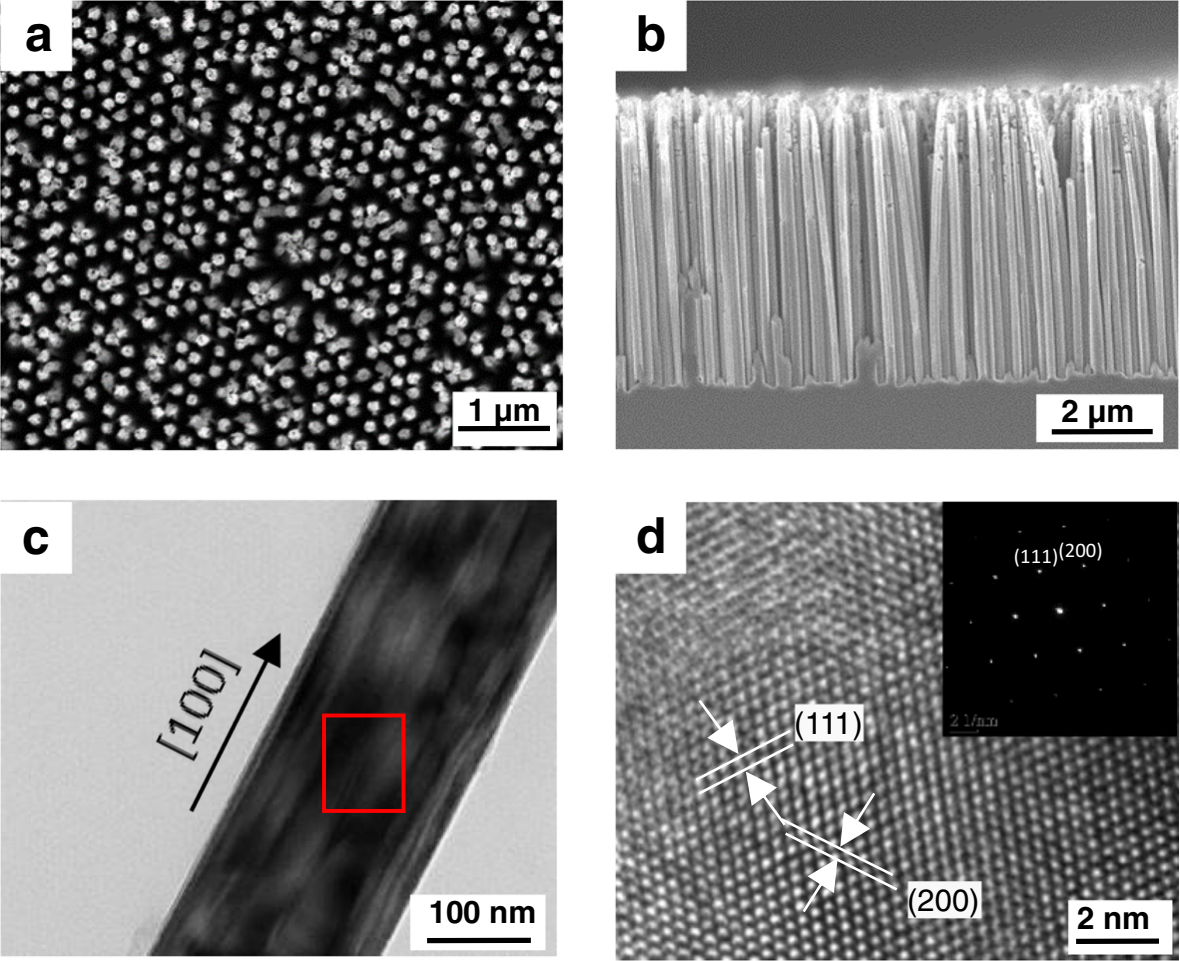
**a** Top-view and **b** cross-sectional SEM images of Si NW arrays fabricated by MACE combined with NSL. **c** TEM image of as-synthesized Si NW. **d** Atomic resolution TEM image of as-synthesized Si NW corresponds to the red square in **c**. The inset is the SAD patterns of Si NW

Figure 2a, b show the plan view and cross-section SEM images, respectively, of the Si NW arrays via MACE combined with NSL. The Si NWs with uniform geometry have diameters between 150 and 200 nm and lengths between 5 and 6 um. Figure 2c shows the TEM image of an individual Si NW, which is a single-crystalline structure and has the preferential etched direction of [100] confirmed by the clear lattice image shown in Fig. 2d.

**Fig. 3:**
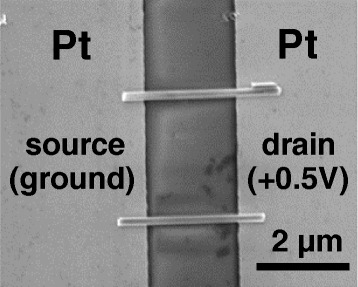
SEM image of parallel-aligned Si NWs across Pt electrodes. A + 0.5 V DC voltage was applied to the drain in the DC-DEP alignment

**Fig. 4:**
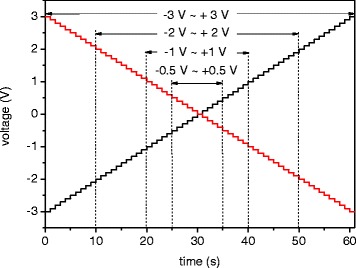
The voltage sweeping rate for the drain electrode by sweeping from negative to positive bias (black line) and from positive to negative bias (red line)

**Fig. 5:**
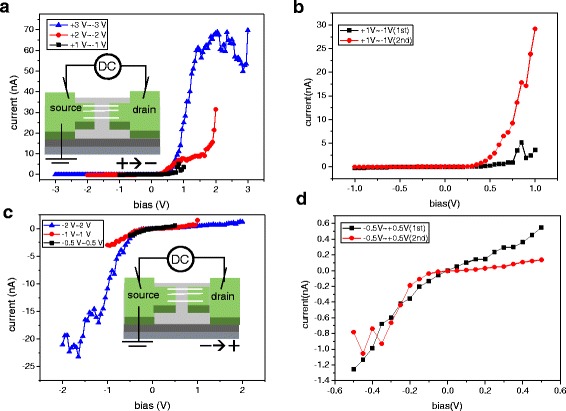
Electrical properties of the parallel Si NWs across Pt electrodes. **a** I-V curves of the parallel Si NWs when a voltage was applied to the drain electrode by sweeping from positive to negative bias, as shown in the inset. There are 24 parallel NWs across Pt electrodes. **b** The I-V curves measured on the first sweep (black line) and second sweep (red line) from + 1 to − 1 V. **c** I-V curves of the parallel Si NWs when a voltage was applied to the drain electrode by sweeping from negative to positive bias, as shown in the inset. There are 18 parallel NWs across Pt electrodes. **d** The I-V curves measured on the first sweep (black line) and second sweep (red line) from − 0.5 to + 0.5 V

In order to study the electrical transport of Si NWs, silicon nanowire-based devices in metal-nanowire-metal structures were fabricated as a + 0.5 V DC was applied to the drain electrode in the DEP aligning process. The top view of the Si NW alignment across the Pt electrodes can be clearly seen from the SEM image, as shown in Fig. 3, where Si NWs are parallel to each other. The density of the aligned Si NWs can be controlled through the concentration of NWs in IPA solution. Two different methods were used to measure the electrical properties of the devices. For method 1 measurement, the I-V curves were measured as the voltage was applied to the drain electrode by sweeping from positive to negative. For method 2 measurement, the I-V curves were measured as the voltage was applied to the drain electrode by sweeping form negative to positive. Surprisingly, the devices have rectifying behavior and the direction of rectification could be determined by a voltage sweep direction as shown in Additional file 1: Figure S1. In order to understand this phenomenon, the I-V curves were measured by applying different ranges of sweep voltage to the drain electrode of the devices. The sweeping rate is shown in Fig. 4. Figure 5a shows that the I-V curves were measured as the voltage was applied to the drain electrode by sweeping from + 1 to − 1 V, + 2 to − 2 V, and + 3 to − 3 V in sequence, as illustrated in the inset of Fig. 5a. It shows rectifying behavior more obviously as the device was measured in the wide voltage range. In Fig. 5b, the I-V red curve was further measured when the voltage was applied to the drain electrode by sweeping from + 1 to − 1 V for the second time. The forward current was 9.2 nA at 0.75 V; the reverse current was around 0.044 nA. The on-to-off current ratio is about 200. It was found that the device became more rectifying compared with the I-V black curve, which was earlier measured in the same voltage range as shown in Fig. 5a with the on-to-off current ratio of 7.7. The opposite rectifying I-V curve can be produced as well when the voltage was applied to the drain electrode by sweeping from − 0.5 to + 0.5 V, − 1 to + 1 V, and − 2 to + 2 V in sequence, as illustrated in the inset of Fig. 5c. It also shows more obvious rectifying behavior in the larger sweep voltage range shown in Fig. 5c. In Fig. 5d, the I-V red curve was further measured when the voltage was applied to the drain electrode by sweeping from − 0.5 to + 0.5 V for the second time. The transition from non-rectifying to rectifying behavior can be observed by comparison with the I-V black curve, which was earlier measured in the same voltage range shown in Fig. 5c. The above I-V characteristic curves suggest that the rectifying behaviors in the Si NW-based devices were produced in the process of the electrical measurement instead of DEP alignment. Furthermore, it was also found that the direction of rectification can be determined by the voltage sweep direction. After the transition from non-rectifying to rectifying, the device had the same rectifying direction no matter what the voltage sweep direction was.

**Fig. 6:**
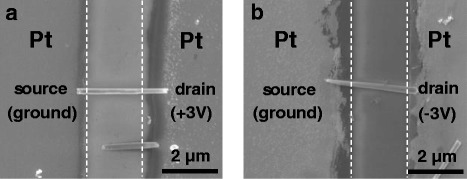
**a** SEM image of Si NW-based device after a + 3 V DC voltage was applied to the drain electrode for Si NW alignment. **b** SEM image of Si NW-based device after a − 3 V DC voltage was applied to the drain electrode for Si NW alignment. The white dash lines show the electrode edge before DEP aligning process

In addition, the zigzag-like I-V curve can be clearly seen when the voltage was applied to the drain electrode by sweeping from + 3 to − 3 V and − 2 to + 2 V, as shown in Fig. 5a, c, respectively. This phenomenon can be explained by the asymmetric Joule heating effects, which is originated from the electric currents flowing through Si NWs as the voltage applied across Pt electrodes is increased. The asymmetric Joule heating effects occur from the uneven distribution of temperature between the electrodes, and the temperature on the anode region is higher than the cathode region [26]. For I-V curve measurement, the current at 3 V applied voltage is about several to hundred nanoamperes as shown in Fig. 5 and Additional file 1: Figure S1, which is much smaller than that in ref. [26]. However, the diameter of Si NWs is about 100 nm, which is much smaller than the width of the channel of the device in ref. [26]. In addition, because the nanowires just adsorbed on the electrodes by the DEP aligning method, the contact area may be much smaller than the cross section of nanowires. Thus, the current density at the NW-electrode contacts may be high enough to cause Joule heating. This can also be seen after + 3 and − 3 V DC were applied to the drain electrode for Si NWs DEP alignment as shown in Fig. 6a, b, respectively. Both figures indicate that the anode regions were severely destroyed by melting compared with the cathode regions.

When the I-V curves of the device were measured in reducing atmosphere (H_2_/Ar), the rectifying property was not obtained by sweeping in the large voltage range (from − 3 to 3 V) as shown in Additional file 2: Figure S2(a). The I-V curve is symmetrical and near linear, which indicates just a small barrier at the interface between the nanowire and two electrodes. However, the Pt and n-Si can theoretically form a Schottky barrier at the Pt/n-type Si contact as the work function of Pt (~ 6.1 eV) is larger than n-type Si (~ 4.15 eV). In this study, the nanowires just adsorb on the electrodes by the DEP aligning method. Thus, the change of barrier height may be due to the gas adsorption on the Si surface. After sweeping in the large voltage range, the slope of I-V curve increased as shown in Additional file 2: Figure S2(b), which indicates that a large voltage range sweeping measurement in reducing gas can reduce the resistance on both NW-electrode contacts. However, air containing O_2_ and H_2_O is an oxidative atmosphere. In air, the oxidation rate of Si is higher at high temperature compared with that at low temperature. Thus, we can infer that for the large voltage range sweeping measurement in air, the increase of barrier height at the anode region is due to the formation of a thin oxidized SiO_*x*_ layer at the interface, which exhibits electron trapping sites.

**Fig. 7:**
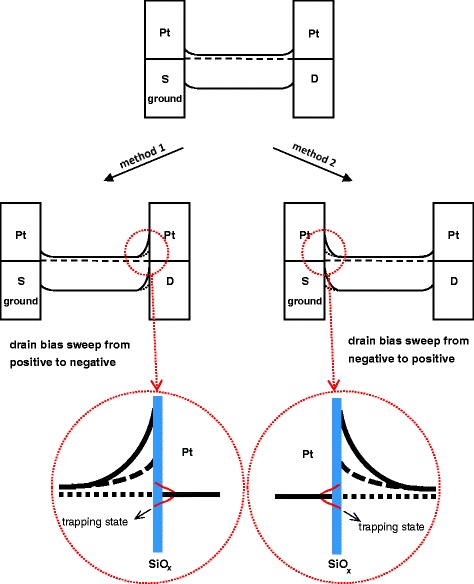
Schematic energy band diagrams for n-type Si/Pt contacts after DC-DEP alignment (top center). The device went through the asymmetric Joule heating process as the voltage was applied to the drain electrode by sweeping from positive to negative bias in method 1 (left) or negative to positive bias in method 2 (right)

Figure 7 shows the schematic energy band diagrams for Si NW-based device before and after the asymmetric Joule heating treatments. Initially, the Pt and n-Si form small equal barrier heights on both ends of the NWs after the DEP alignment. When the voltage was applied to the drain electrode by sweeping from positive to negative (in method 1) or negative to positive bias (in method 2), the barrier height on the high-temperature anode side would be tuned simultaneously due to the asymmetric Joule heating effects. In other words, the barrier height would be increased and dominate the rectifying behavior of the device as we deduce from the rectifying I-V characteristics shown in Fig. 5.

**Fig. 8:**
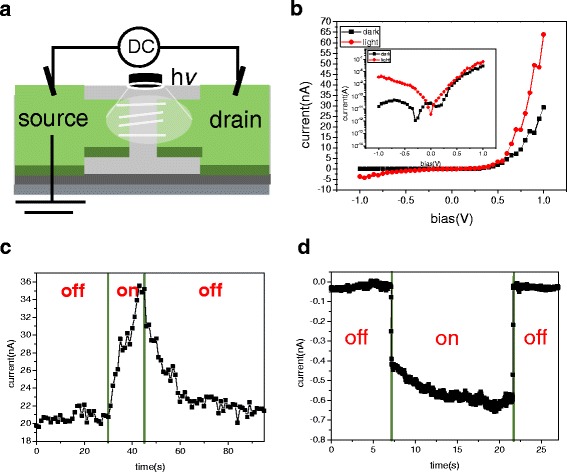
**a** Schematic of a Si NW-based sensor under white light illumination with the intensity of 825 mW/cm^2^. **b** I-V curves of a Si NW-based sensor under the dark and white light illumination. The inset shows the corresponding I-V properties in a semi-logarithmic scale. **c** Time-resolved photoresponse of a Si NW-based sensor at + 0.75 V in forward-biased mode under white light illumination by switching on and off. **d** Time-resolved photoresponse of a Si NW-based sensor at − 0.75 V in reverse-biased mode under white light illumination by switching on and off. The prepared device was the same as that for conduct electrical transport property measurement in Fig. 5a, b

To investigate the photosensing properties of the rectifying Si NW-based device in this case, a broadband white light with the intensity of 825 mW/cm^2^ was vertically shown on the device while the corresponding photoresponse characteristics were recorded, as depicted in Fig. 8a. Figure 8b shows the I-V curves of this device under dark (black curve) and broadband white light irradiation (red curve). It reveals that the photocurrent could be induced, and the higher sensitivity was achieved when the device exhibited reverse I-V characteristics shown in the inset of Fig. 8b. The time-dependent photoresponse behavior was investigated when the device was exposed to the white light by switching on and off. As shown in Fig. 8c, where the device was under a white light excitation at + 0.75 V in forward-biased mode, the current increased from 20 to 35 nA within 15 s, which is improved for only 75%. When the white light was turned off, the current decreased to the initial value within 30 s. On the other hand, when the device was under a white light excitation at − 0.75 V in reverse-biased mode, as shown in Fig. 8d, the current increased abruptly from 40 to 430 pA within 64 ms, which is up to 13 times larger than the device in forward-biased mode. Furthermore, the higher recovery rate can be observed as the current decreased to the initial value from the saturation state within only 48 ms at the moment when the white light was off.

In terms of the photoresponse performance, the discrepancy of these above results can be explained as follows. When the device is in forward-biased mode, the depletion region width decreases and enhances the current flow that leads to the lower sensitivity to white light. However, the device in reverse-biased mode, by contrast, has the larger depletion region where the strong built-in electric field exists. The photogenerated electrons and holes can be separated efficiently and reduce the electron-hole recombination rates under the white light illumination, thus resulting in an abrupt increase in free carrier density. Therefore, rectifying devices have a high response rate property. However, in previous studies [27, 28], rectifying devices with one Ohmic contact electrode and the other Schottky contact electrode were fabricated by selecting various electrode materials. In this study, an easy manufacturing process was used. The rectifying behavior of the NW devices formed by dielectrophoresis alignment was obtained just by asymmetric Joule heating in the electrical measurement process.

## Conclusions

In summary, the Si NW-based devices were fabricated by aligning the single-crystalized Si NWs across the Pt electrodes using DC-DEP method. The rectifying I-V characteristics of these devices can be obtained, and the direction of rectification can be determined by the voltage sweep direction. This phenomenon can be associated with the asymmetric Joule heating effects produced in the electrical measurement process. The high speed and high photoresponse can be achieved for the rectifying devices in reverse-biased mode due to the efficient electron-hole separation by strong built-in electric field in the depletion region. This rectifying Si NW-based device can potentially be used for photodetectors and other applications such as logic gates or sensors.

## Additional files

Additional file 1: **Figure S1.** The I-V curves were measured in large sweep voltage range. (PDF 154 kb)

Additional file 2: **Figure S2.** Electrical properties of the parallel Si NWs across Pt electrodes in reducing atmosphere (H_2_/Ar). (PDF 219 kb)
