# Supplementary material for: Effects of Asymmetric Local Joule Heating on Silicon Nanowire-Based Devices Formed by Dielectrophoresis Alignment Across Pt Electrodes
Source: Nanoscale Res Lett. 2018 Jan 16;13:21. doi: 10.1186/s11671-017-2423-z (PMC5773461; doi:10.1186/s11671-017-2423-z)
Supplement: Supplementary file 1 — Figure S1. The I-V curves were measured in large sweep voltage range. (PDF 154 kb) [file 11671_2017_2423_MOESM1_ESM.pdf]

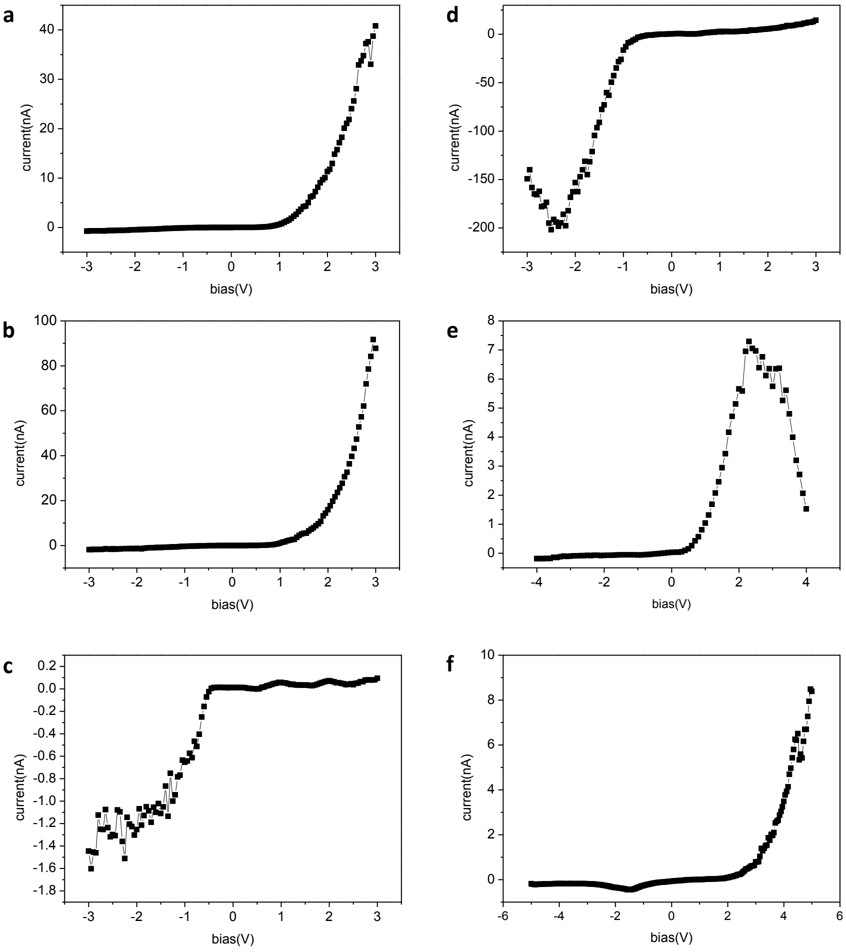

Figure S1 The I-V curves measured in large sweep voltage range. The I-V curves measured as the voltage was applied to the drain electrode by sweeping from **a**,**b**+3 V to -3 V, **c**,**d** -3 V to +3 V, **e** +4 V to -4 V and **f** +5 V to -5 V. The devices were fabricated by applying a **b**-**e** -0.5 and **a**,**f** -0.5 V DC to the drain electrodes in the DEP aligning process. The numbers of parallel NWs across Pt electrodes are 6, 6, 4, 18, 4 and 27 for **a**, **b**, **c**, **d**, **e** and **f**, respectively.
