# Supplementary material for: Effects of Asymmetric Local Joule Heating on Silicon Nanowire-Based Devices Formed by Dielectrophoresis Alignment Across Pt Electrodes
Source: Nanoscale Res Lett. 2018 Jan 16;13:21. doi: 10.1186/s11671-017-2423-z (PMC5773461; doi:10.1186/s11671-017-2423-z)
Supplement: Supplementary file 2 — Figure S2. Electrical properties of the parallel Si NWs across Pt electrodes in reducing atmosphere (H2/Ar). (PDF 219 kb) [file 11671_2017_2423_MOESM2_ESM.pdf]

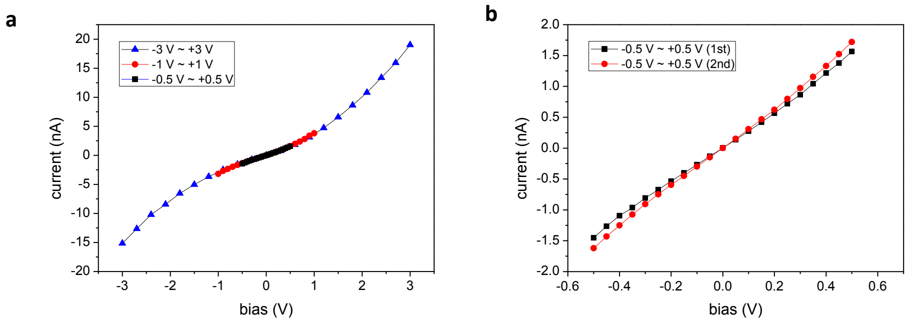


Figure S2 Electrical properties of the parallel Si NWs across Pt electrodes in reducing atmosphere ($H_2$/Ar). **a** I-V curves of the parallel Si NWs when a voltage was applied to the drain electrode by sweeping from negative to positive bias. There are 5 parallel NWs across Pt electrodes. **b** The I-V curves measured on the first sweep (black line) and second sweep (red line) from -0.5 V to + 0.5 V.
